# Assessing the association between antibody status and symptoms of long COVID: A multisite study

**DOI:** 10.1371/journal.pone.0304262

**Published:** 2024-06-06

**Authors:** Ingrid A. Binswanger, Darryl E. Palmer-Toy, Jennifer C. Barrow, Komal J. Narwaney, Katia J. Bruxvoort, Courtney R. Kraus, Jason A. Lyons, Jessica A. Lam, Jason M. Glanz

**Affiliations:** 1 Institute for Health Research, Kaiser Permanente Colorado, Aurora, Colorado, United States of America; 2 Colorado Permanente Medical Group, Aurora, Colorado, United States of America; 3 Department of Medicine, University of Colorado School of Medicine, Aurora, Colorado, United States of America; 4 Bernard J. Tyson School of Medicine, Pasadena, California, United States of America; 5 Southern California Permanente Medical Group Regional Reference Laboratories, North Hollywood & Chino Hills, California, United States of America; 6 Department of Epidemiology, University of Alabama at Birmingham, Birmingham, Alabama, United States of America; 7 Department of Clinical Analysis at Southern California Permanente Medical Group, California, CA, United States of America; 8 Department of Epidemiology, Colorado School of Public Health, Aurora, Colorado, United States of America; Universidade Federal do Para, BRAZIL

## Abstract

The association between SARS-CoV-2 humoral immunity and post-acute sequelae of COVID-19 (long COVID) remains uncertain. The objective of this population-based cohort study was to assess the association between SARS-CoV-2 seropositivity and symptoms consistent with long COVID. English and Spanish-speaking members ≥ 18 years old with SARS-CoV-2 serologic testing conducted prior to August 2021 were recruited from Kaiser Permanente Southern California and Kaiser Permanente Colorado. Between November 2021 and April 2022, participants completed a survey assessing symptoms, physical health, mental health, and cognitive function consistent with long COVID. Survey results were linked to SARS-CoV-2 antibody (Ab) and viral (RNA) lab results in electronic health records. Weighted descriptive analyses were generated for five mutually exclusive patient groups: (1) +Ab/+RNA; (2) +Ab/- or missing RNA; (3) -Ab/+RNA; (4a) -Ab/-RNA reporting no prior infection; and (4b) -Ab/-RNA reporting prior infection. The proportions reporting symptoms between the +Ab/+RNA and -Ab/+RNA groups were compared, adjusted for covariates. Among 3,946 participants, the mean age was 52.1 years old (SD 15.6), 68.3% were female, 28.4% were Hispanic, and the serologic testing occurred a median of 15 months prior (IQR = 12–18). Three quarters (74.5%) reported having had COVID-19. Among people with laboratory-confirmed COVID-19, there was no association between antibody positivity (+Ab/+RNA vs. -Ab/+RNA) and any symptoms, physical health, mental health, or cognitive function. As expected, physical health, cognitive function, and fatigue were worse, and palpitations and headaches limiting the ability to work were more prevalent among people with laboratory-confirmed prior infection and positive serology (+Ab/+RNA) compared to those without reported or confirmed prior infection and negative serology (-Ab/-RNA/no reported COVID-19). Among people with laboratory-confirmed COVID-19, SARS-CoV-2 serology from practice settings were not associated with long COVID symptoms and health status suggesting limited utility of serology testing for long COVID.

## Introduction

With much of the population having been infected with SARS-CoV-2, complications of COVID-19 may affect millions of global survivors, resulting in lost economic productivity, quality of life reductions, functional limitations, and disability [1–7]. Chronic post-acute sequelae of COVID-19 (PASC, also called “long COVID”) involves multisystemic symptoms, signs, and health conditions that develop during or following a confirmed or suspected COVID-19 case, that may be persistent, recurrent, or new [8–11]. Common symptoms include dyspnea, fatigue, insomnia, joint and muscle pain, and anosmia [6,12–17], with prevalence estimates varying across symptoms, severity of the initial infection, and population studied [18–22].

The U.S. Food and Drug Administration approved the first serologic tests to detect antibodies against SARS-CoV-2 under Emergency Use Authorization in April 2020. Since then, guidelines from the U.S. Centers for Disease Control and Prevention and the Infectious Diseases Society of America have suggested a limited clinical role for SARS-CoV-2 antibody testing, with positive antibodies suggesting prior infection (or vaccination for anti-spike protein tests) or supporting a diagnosis of multisystem inflammatory syndrome [23,24]. Despite this narrow role, there has been interest in whether antibody test results are associated with or can predict long COVID [22]. For instance, a study of 146 hospitalized patients demonstrated that lower anti-spike IgG peak titer was associated with a higher burden of long COVID symptoms [25]. It has also been postulated that spike protein could cause direct damage to the blood-brain barrier, leading to neurologic complications [26], in which case anti-spike antibodies could protect from cognitive symptoms of long COVID [27].

Finding an association between serology results and long COVID symptoms could inform mechanistic pathways, guide predictive and prognostic models, and support new indications for serology testing. Thus, this study’s objective was to assess the association between SARS-CoV-2 serology results and long COVID symptoms and health domains in California and Colorado health systems.

## Methods

### Study design and study settings

We conducted a population-based cohort study of adults who received commercially available serologic tests for SARS-CoV-2 antibodies to assess how mutually exclusive combinations of serologic and molecular results, supplemented with survey data on prior COVID-19, were associated with symptoms and health domains consistent with long COVID (S1 Fig in S1 File). The survey was conducted in Kaiser Permanente Southern California (KPSC) and Colorado (KPCO) integrated health care delivery systems, which comprise employer-based and individual insurance, and Medicare and Medicaid plans. KPSC serves approximately 4.8 million members and KPCO serves approximately 0.5 million members. For context in these study settings, the first California and Colorado COVID-19 cases were identified in January and March of 2020, respectively. California experienced a peak of Alpha variant mid-May 2021, Delta from June to mid-December 2021, and Omicron starting in late December 2021. Alpha peaked in Colorado prior to May, Delta from May to December, and Omicron after December 2021 [28]. Vaccines (Pfizer-BioNTech BNT162b2, Moderna mRNA-1273, and Janssen JNJ-7846725) became available in December 2020. This study was conducted as part of a multisite collaboration to assess the clinical roles of serology across our health systems [29,30].

For varied indications (e.g., patient request, physician order for symptoms, research studies, community events), clinical staff collected nasopharyngeal, anterior nares, saliva, or oropharyngeal specimens for SARS-CoV-2 molecular testing, conducted phlebotomy for serology, and conducted laboratory assays in each health system. The health systems employed the Abbott anti-nucleocapsid IgG test, Roche anti-nucleocapsid total IgG, and three anti-spike receptor binding domain assays (Siemens ADVIA Centaur SARS-CoV-2 Spike Total IgG/IgM, Spike IgG COV2G, and sCOVG Spike IgG) [31,32]. Anti-spike antibodies can be induced by natural infection or vaccination, while anti-nucleocapsid antibodies are induced only by infection. SARS-CoV-2 molecular (RNA) testing results were derived from real-time reverse transcription polymerase chain reaction assays made by Roche (Rotkreuz, Switzerland) and ThermoFisher (Waltham, MA), and transcription mediated amplification assay for the qualitative detection of SARS-CoV-2 by Aptima on the Panther (Hologic, Inc., San Diego, CA). A positive test was defined in accordance with the manufacturer’s instructions for each test.

Both health systems maintained comprehensive electronic health record (EHR) databases, with details from health care encounters, including diagnoses, procedures, laboratory tests and results, pharmacy records, and vaccination data. Information from state immunization registries were also available. Data were coded according to a common data model to facilitate extraction using distributed code.

### Participant recruitment

Eligible participants were KPSC and KPCO members ≥18 years with ≥1 SARS-CoV-2 serology result before the study start date (August 4, 2021) who spoke English or Spanish, had not opted out of research, and had an available contact method. If members had >1 serology test, the index test was the positive serology result most proximal to August 4, 2021; if no positive result was identified, the index test was the most proximal negative result. Serologic results on or after the first COVID-19 vaccine dose were excluded. Eligible members had at least 12 months of enrollment in a KP health plan prior to the index serology test to capture covariates. Members with a negative serology test and no RNA tests were excluded as COVID-19 could not be confirmed. For members with multiple RNA results before the index serology test date, the most proximal to the index serology was designated as the “index” result, selecting positive RNA test results over negative, and negative over missing results. For positive index serologies, the RNA test prior to, or on the day of, the positive serology test, was selected. To classify serology as negative, we required ≥14 days between the index serology and the RNA test to ensure adequate time elapsed to seroconvert. To be classified as RNA negative, all RNA tests prior to the index serology had to be negative.

*A priori*, we created four exposure groups based on the index serology test and RNA tests drawn before the index serology test date (Table 1). Among serology-positive participants, the Ab+/RNA+ group (1) was considered to have had SARS-CoV-2 infection with an expected immune response whereas the Ab+/RNA- or RNA missing group (2) was considered to have had a false-positive serology or not have been tested by SARS-CoV-2 RNA due to less severe infection. Among serology-negative participants, the Ab-/RNA+ group (3) was presumed to have an impaired or waning immune response whereas the Ab-/RNA- group (4) was planned as a control group. However, the majority of participants in the Ab-/RNA- group reported prior COVID-19 in the survey (described below), so this group was subdivided into an Ab-/RNA-/no prior COVID-19 group consistent with a “true control” group (4a) and an Ab-/RNA-/prior COVID-19 group consistent with prior self-reported COVID-19 (4b).

**Table 1:** COVID-19 test groups.

Test Group	Serology test results	RNA test results	Survey results on past COVID-19	Interpretation	No. of study participants
1	≥1 positive	≥1 positive	Any response	Infected with SARS-CoV-2 with evidence of an expected immune response	1537
2	≥1 positive	None or all negative	Any response	Not tested by RNA for SARS-CoV-2 (due to less severe infection or false-positive serology)	1338
3	All negative	≥1 positive	Any response	Presumptive impaired or waning immune response	379
4a	All negative	All negative	Reported no prior infection^a^	Control	302
4b	All negative	All negative	Reported prior infection^a^	Self-report (without record of positive RNA test in EHR data)	390

Abbreviation: EHR = electronic health record data.

^a^Based on the question: “Did you have, or think you had, COVID virus infection?”.

Eligible members in each health system were selected using stratified random sampling technique by the study site, four exposure groups, and language. All eligible Spanish-speaking members were sampled from all groups. All eligible English-speaking members in the Ab+/RNA+ and Ab-/RNA+ groups at KPCO and Ab-/RNA+ group at KPSC were sampled to achieve target sample sizes.

Statistical power was calculated for *a priori* comparisons in any dyspnea between the Ab+/RNA+ vs. Ab-/RNA+ groups and Ab+/RNA+ vs. Ab-/RNA- groups. The assumed power was 80%, with an alpha of 0.05, design effect of 2, and prevalence of dyspnea of 25% and 15% for groups 3 and 4 respectively. The sample sizes needed for a difference in proportions of 0.103 between the Ab+/RNA+ vs. Ab-/RNA+ groups and 0.071 between Ab+/RNA+ vs. Ab-/RNA- groups were 1500 for Ab+/RNA+ group, 400 for Ab-/RNA+ group, and 700 for the Ab-/RNA- group.

### Survey development and content

The investigators developed a survey instrument to measure 31 potential long COVID symptoms and health domains, based on conditions and symptoms reported to be associated with long COVID, with existing, validated scales for quality of life, gastrointestinal symptoms, anxiety/depression, cognition, pain, dyspnea, sleep, and fatigue from the Patient-Reported Outcomes Measurement System (PROMIS); chest pain from the Seattle Angina Questionnaire; palpitations (including tachycardia, heart flutters, and extra or missed heart beats) from the Health and Quality of Life Outcomes for Arrythmia; headaches limiting ability to work from the ID-Migraine Questionnaire; fainting/lightheadedness from the Scales for Outcomes in Parkinson’s Disease Autonomic Dysfunction; sense of smell and taste from the University College of London Patient Led Research Questionnaire on Long COVID; hair loss from the Hamilton-Norwood and Ludwig scales; and newly developed items for menstrual symptoms (S1 Table in S1 File) [33–39]. To maximize recall, items referred to the last 7 days, except hair loss, lightheadedness, headaches, and chest pain, which referred to the last 4 weeks, and menstrual symptoms, which referred to the last 3 months. For participants who reported prior COVID-19, a checklist was developed for 31 symptoms (e.g., fatigue, brain fog, body aches) that were persistent (≥ 3 months), recurrent (went away for ≥ 2 weeks and then returned) or new (occurred ≥ 2 months after having had COVID-19). The survey also assessed race, ethnicity, gender, whether participants had or thought they had prior COVID-19, hazardous drinking using the Alcohol Use Identification Test (AUDIT-C) [40], current tobacco use or vaping [41,42], and vaccination status. The survey was professional translated into Spanish and pre-tested by a multidisciplinary clinical and research group, survey researchers, and a physician who treats long COVID followed by a pilot test with 100 members. Surveys were administered electronically using Research Electronic Data Capture (REDCap) [43] and on paper.

### Recruitment and survey procedures

Initial recruitment occurred ≥ 12 weeks after serology testing by email and text messaging with a link to a REDCap face page containing information about the study and a link to the survey. After electronic recruitment attempts, one recruitment attempt was made by US mail. Spanish surveys were provided if the EHR indicated Spanish as a preferred language. Recruitment occurred between November 18, 2021, and April 20, 2022, and data collection closed at the end of June 2022. Participants were renumerated $10. Participants were contacted to verify if key information about outcomes were missing; participants with non-response or missing data to prior COVID-19, symptom and health domain measures, or key covariates were excluded from the final study population.

### Ethics approval and study reporting

The KPSC Institutional Review Board approved the study and waived the requirement for written informed consent due to minimal risk to study participants. Potential participants received information about the study and agreed to the use of survey answers and electronic health record information. Study reporting follows the Strengthening the Reporting of Observational Studies in Epidemiology checklist.

### Data analysis

Variables extracted from the EHR included age, sex, language preference, insurance enrollment, RNA and serology test dates, test results, and vaccination dates. We compared characteristics of participants and non-respondents using EHR data. Data quality was assessed by examining missing data, congruence between reported prior COVID-19 and RNA results, and timing of reported COVID-19 relative to COVID-19 surges in each region.

After accounting for the stratified sampling design and applying weights to account for sampling and survey response probabilities, we described self-reported clinical and demographic participant characteristics using weighted percentages, means (SD) and standard errors, such as the proportions with prior COVID-19 and persistent, recurring, and new symptoms. For PROMIS measures, raw scores were summed, reverse scored when appropriate, and converted to standardized T scores in which a score of 50 represents the mean score of a reference population (i.e., the US general population), with an SD of 10. For PROMIS measures with previously defined general population cut-offs, those were applied to identify higher levels of impairment in the outcomes [34]. When published cut-offs were only derived from clinical samples (e.g., patients with gastrointestinal conditions), the 75^th^ percentile was the cut-off. For measures with no published cut-offs, any reported symptom was the cut-off. For measures asked as a single question, the dichotomized responses were used as the outcome. Multivariable logistic regression was applied, accounting for the sampling strategy and including the health system, age, sex, race, ethnicity, and time between serology test date and survey date as covariates. Separate models were run for each measure as the outcome. To assess our hypothesis that antibody presence was associated with long COVID among people with a history of COVID-19 infection, we compared group 1 (Ab+/RNA+) to group 3 (Ab-/RNA+). To check the validity of our data, we assessed whether people who had COVID-19 with an expected immune response would report more impairment than people who had not had COVID-19 by comparing group 1 (Ab+/RNA+) to group 4a (true controls, Ab-/RNA-/no prior reported COVID-19).

### Secondary and sensitivity analyses

In secondary analyses, group 1 (Ab+/RNA+) was compared to group 2 (Ab+/RNA-) and participants who were hospitalized with their initial infection were compared to those who were not. In the overall sample, we also examined the physical and mental health by vaccine status. In sensitivity analyses, we applied linear regression to examine T scores as continuous variables for measures with general population estimates [34], comparing group 1 (Ab+/RNA+), group 3 (Ab-/RNA+), and group 4a (true control group). Due to waning antibody levels over time [44,45], we examined the association between antibody status and fair/poor physical health and fair/poor mental health for individuals in which the time between the RNA test and the antibody test was 3 or fewer months. Finally, we assessed the association between antibody status and symptoms adjusting for the modified Charlson co-morbidity score assessed in the year prior to the index serology test date [46]. Otherwise, secondary and sensitivity analyses adjusted the same covariates as the primary analyses.

## Results

**Fig 1:**
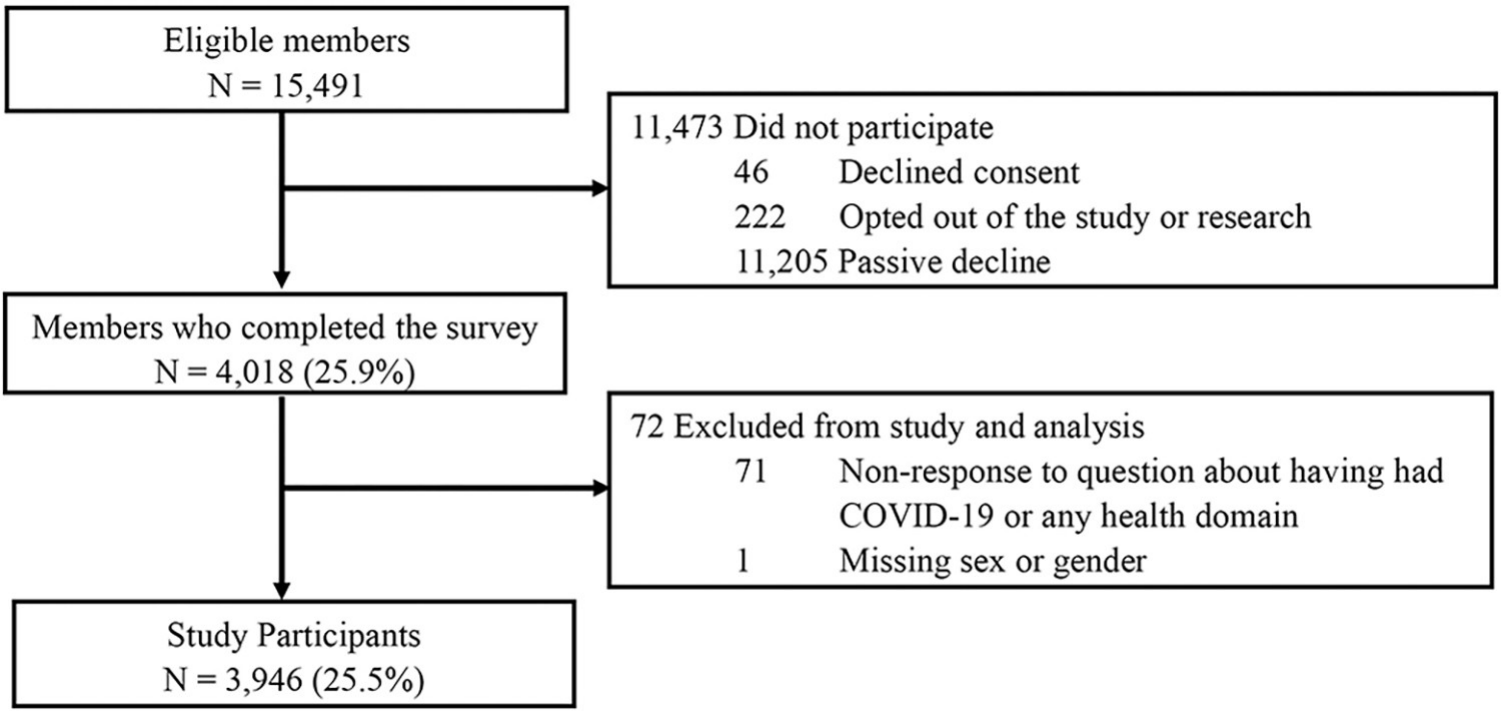
Study flow diagram.

Of 15,491 recruited members (KPSC: 11,023; KPCO: 4,468), 4,018 (25.9%) members completed the survey, and 3,946 (25.5%) represented the final study population (Fig 1). Participation rates differed by site, and there were differences between respondents and non-respondents by language, recruitment group, and demographics in the EHR (S2 Table in S1 File). Eligible index RNA tests were unavailable for 14.5% of participants. The mean age of participants was 52.1 years (SD = 15.6) and 4.4% (n = 173) completed the survey in Spanish and 33.4% (n = 1317) by paper. The median time between PCR and serology was approximately 2 months (median = 59 days, range 1–470; IQR 29–113) and the median time between serology and survey completion was over a year (median = 446 days, range 121–773; IQR 366–542).

Table 2 indicates how participants identified their gender, race, ethnicity, sexual orientation, education, and vaccine status. Using weighted survey responses, 17.6% met criteria for hazardous drinking, 5.6% smoked tobacco and/or vaped, 11.0% had not been immunized against COVID, and 74.5% reported prior COVID-19, of whom 8.3% were hospitalized during their illness. The timing of prior COVID-19 aligned with peaks of variant activity (S2 Fig in S1 File). Small discrepancies between lab testing and self-report were identified, such as 6.1% of participants in the Ab+/RNA+ group who reported no prior COVID-19. The majority (58.1%) of Ab-/RNA- participants reported they had prior COVID-19 and were assigned to group 4b rather than the true control group.

**Table 2:** Self-reported participant demographic and clinical characteristics by groups.

	Weighted percentage (SE)					
Characteristic	Overall	(1) Ab+/RNA+	(2) Ab+/RNA- or no RNA test	(3) Ab-/RNA+	(4a) Ab-/RNA-/no prior COVID-19^a^	(4b) Ab-/RNA-/prior COVID-19^a^
**Gender**						
Female	69.6 (1.0)	65.9 (1.3)	68.2 (1.3)	74.3 (2.0)	72.0 (2.9)	71.4 (2.6)
Male	29.5 (1.0)	33.3 (1.3)	31.2 (1.3)	23.8 (2.0)	27.1 (2.9)	27.4 (2.5)
Transgender/Non-binary/other gender	0.3 (0.1)	0.2 (0.1)	0.1 (0.0)	0.5 (0.3)	0.8 (0.6)	0
Rather not answer	0.7 (0.2)	0.6 (0.2)	0.5 (0.2)	1.4 (0.5)	0	1.2 (0.7)
**Race**						
American Indian/AK Native	1.1 (0.3)	1.0 (0.3)	0.9 (0.3)	0.6 (0.4)	1.1 (0.7)	1.5 (0.7)
Asian/Pacific Islander	9.3 (0.7)	10.1 (0.9)	8.1 (0.8)	8.3 (1.3)	10.1 (2.1)	9.0 (1.7)
Black/African American	3.9 (0.4)	4.2 (0.6)	4.8 (0.6)	2.9 (0.8)	4.7 (1.5)	2.4 (0.9)
White	62.8 (1.1)	57.0 (1.4)	62.1 (1.3)	65.1 (2.2)	70.3 (3.1)	62.8 (2.8)
Multiracial	4.0 (0.4)	3.9 (0.6)	5.2 (0.6)	5.0 (1.0)	3.6 (1.3)	3.5 (1.1)
Additional groups	12.7 (0.8)	17.1 (1.1)	11.8 (1.0)	11.7 (1.5)	7.1 (1.8)	13.6 (2.0)
Rather not answer/missing	6.2 (0.5)	6.7 (0.7)	7.1 (0.8)	6.5 (1.2)	3.2 (1.1)	7.3 (1.5)
**Hispanic, Latino/a, or of Spanish origin**						
Not Hispanic/of Spanish origin	56.5 (1.1)	49.1 (1.3)	57.7 (1.3)	60.4 (2.2)	64.1 (3.2)	56.2 (2.8)
Mexican/Mexican American/ Chicano/a	23.0 (1.0)	26.9 (1.3)	22.8 (1.2)	20.1 (1.8)	13.2 (2.2)	27.3 (2.6)
Puerto Rican/Cuban/other Hispanic	9.1 (0.6)	11.5 (0.9)	8.7 (0.8)	7.2 (1.2)	9.4 (2.0)	7.3 (1.5)
Rather not answer/missing	11.4 (0.7)	12.4 (0.9)	10.9 (0.9)	12.3 (1.5)	13.3 (2.3)	9.2 (1.6)
**Sexual orientation**						
Straight or heterosexual	85.2 (0.8)	86.3 (1.0)	86.3 (1.0)	85.8 (1.6)	82.2 (2.6)	85.4 (2.0)
Lesbian or gay	3.8 (0.5)	2.1 (0.4)	3.4 (0.5)	4.0 (0.9)	3.1 (1.2)	6.1 (1.4)
Bisexual	2.1 (0.4)	1.1 (0.3)	2.2 (0.4)	2.7 (0.7)	3.3 (1.3)	2.1 (0.8)
Asexual/something else	0.6 (0.2)	0.1 (0.0)	0.3 (0.1)	0.6 (0.3)	1.2 (0.7)	0.8 (.5)
Rather not answer/missing/do not know	8.3 (0.6)	10.3 (0.9)	7.8 (0.8)	6.9 (1.2)	10.2 (2.1)	5.6 (1.3)
**Education**						
Less than high school	2.4 (0.3)	4.2 (0.5)	2.6 (0.5)	1.1 (0.5)	1.5 (0.6)	1.7 (0.7)
High school or equivalent	12.8 (0.7)	14.5 (1.0)	15.2 (1.0)	9.6 (1.4)	11.6 (2.1)	10.8 (1.8)
Two-year degree or some college	27.3 (1.0)	29.5 (1.3)	27.0 (1.2)	28.5 (2.1)	23.8 (2.8)	27.9 (2.6)
Four-year college degree	28.2 (1.0)	25.7 (1.2)	26.9 (1.2)	27.3 (2.0)	29.1 (3.0)	30.9 (2.6)
Master’s degree	16.0 (0.8)	13.1 (0.9)	17.0 (1.0)	19.7 (1.8)	16.7 (2.3)	16.6 (2.1)
Doctoral degree	5.9 (0.6)	4.7 (0.6)	5.7 (0.6)	5.2 (1.0)	8.2 (1.8)	5.8 (1.3)
Rather not answer/missing	7.4 (0.6)	8.3 (0.8)	5.7 (0.7)	8.5 (1.3)	9.2 (2.0)	6.3 (1.4)
**Smoking or vaping**						
Yes	5.6 (0.5)	3.3 (0.5)	6.0 (0.7)	5.4 (1.0)	6.1 (1.5)	7.0 (1.4)
No	89.8 (0.7)	91.4 (0.8)	90.9 (0.8)	90.4 (1.4)	86.5 (2.3)	89.7 (1.7)
Missing	4.7 (0.5)	5.4 (0.7)	3.2 (0.5)	4.2 (0.9)	7.4 (1.8)	3.3 (1.0)
**Hazardous drinking (AUDIT-C)**						
Yes	17.6 (0.9)	14.4 (0.9)	19.3 (1.0)	15.9 (1.7)	18.7 (2.5)	18.6 (2.1)
No	71.2 (1.0)	72.7 (1.2)	70.1 (1.3)	72.3 (2.1)	68.9 (3.0)	72.0 (2.5)
Missing	11.3 (0.7)	12.9 (1.0)	10.6 (0.9)	11.8 (1.5)	12.5 (2.2)	9.4 (1.7)
**Vaccination status**						
Not vaccinated	11.0 (0.7)	12.2 (0.9)	16.2 (1.0)	14.7 (1.7)	4.8 (1.5)	9.7 (1.8)
Received at least 1 vaccine	23.4 (0.9)	26.2 (1.2)	26.6 (1.3)	34.7 (2.2)	18.2 (2.6)	20.6 (2.3)
Received booster	61.6 (1.1)	57.3 (1.4)	54.2 (1.4)	47.2 (2.3)	70.4 (3.1)	67.1 (2.7)
Missing	4.0 (0.4)	4.2 (0.6)	3.0 (0.5)	3.3 (0.8)	6.6 (1.8)	2.6 (0.9)
**Reported prior COVID** ^**a**^ ^,^ ^**b**^						
Yes	74.5 (0.3)	93.9 (0.7)	86.1 (1.0)	86.0 (1.6)	0	100 (0)
No	25.5 (0.3)	6.1 (0.7)	13.9 (1.0)	14.0 (1.6)	100 (0)	0

^a^Based on the question: “Did you have, or think you had, COVID virus infection?”.

^b^By design, all group 4a participants answered “No” and group 4b participants answered “Yes”.

**Fig 2:**
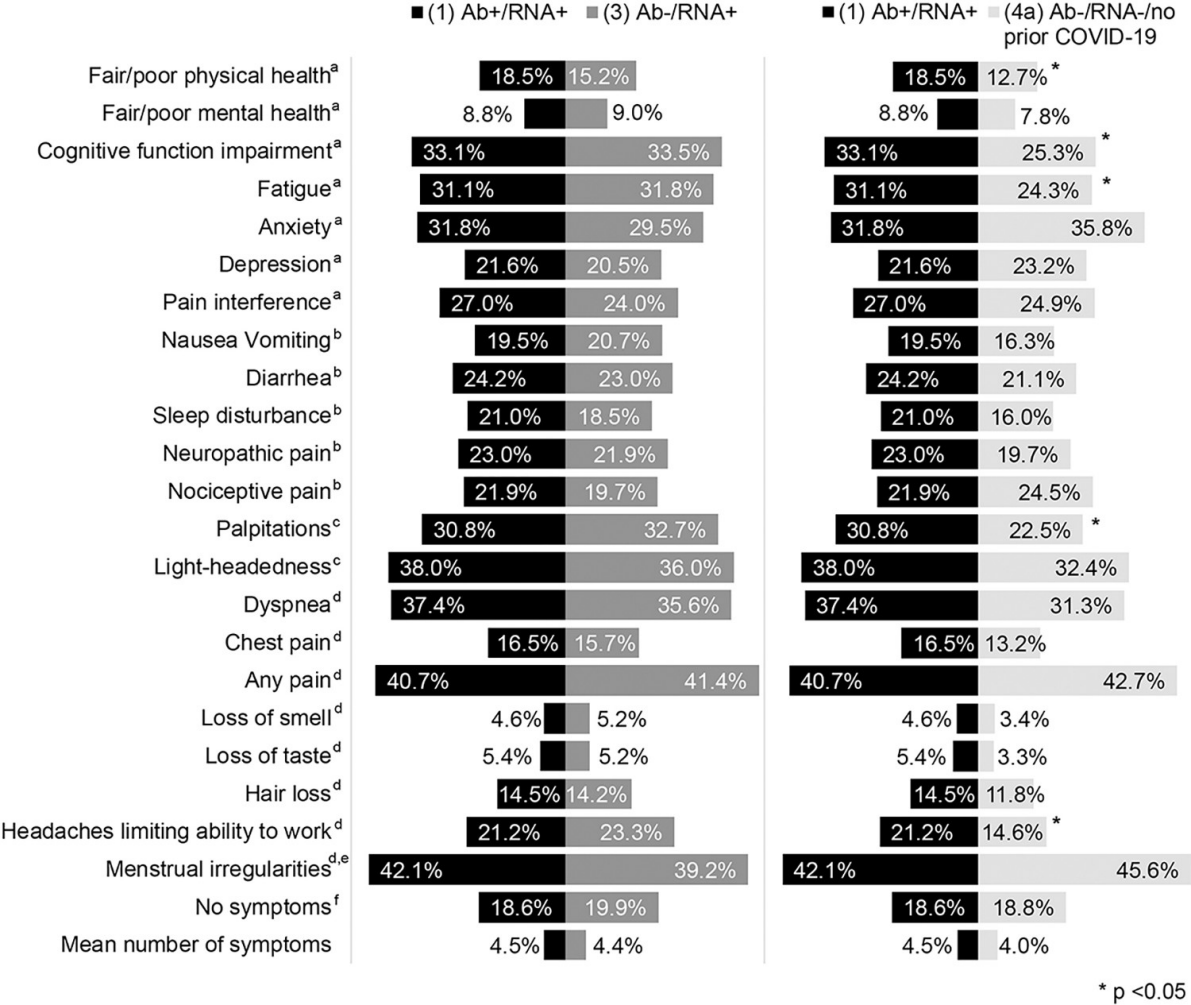
Weighted percentage of participants reporting symptoms by group. ^a^PROMIS T-score (using PROMIS cut-offs): Physical health, mental health: Fair/poor versus good/very good/excellent; cognitive function, fatigue, anxiety, depression, pain interference: Mild/moderate/severe versus normal. ^b^PROMIS T-score (using cut-offs from data): Nausea/vomiting, diarrhea, sleep disturbance, neuropathic pain, nociceptive pain: 75^th^ percentile. ^c^More than 1 question asked, analyzed as present if participants answered yes to any of the questions in that domain. ^d^Single question asked for the domain and analyzed. ^e^Reported in menstruating women. ^f^Reported no symptoms.

Among participants with laboratory-confirmed prior SARS-CoV-2 infection (i.e., RNA+), no significant differences in symptoms, number of symptoms, or health domains were observed by serology status (Fig 2). Just over a third of participants in group 1 (Ab+/RNA+) and group 2 (Ab+/RNA-) reported dyspnea (37.37% vs. 35.57%, p = 0.51). In contrast, group 1 (Ab+/RNA+) compared reported worse physical health, cognitive function, and fatigue, and a higher prevalence of palpitations and headaches affecting work function than the true controls (group 4a).

In secondary analyses, group 1 (Ab+/RNA+) reported worse physical health and more fatigue, diarrhea, sleep disturbance, dyspnea, chest pain, pain interference, neuropathic pain, nociceptive pain, and overall pain compared with the group 2 (Ab+/RNA-) (S3 Table in S1 File). Group 4a (Ab-/RNA- group reporting prior COVID-19) reported more impairment (e.g., mental health) than group 1 (Ab+/RNA+). People who were hospitalized with their initial infection had more severe symptoms than those who did not (S4 Table in S1 File). There was no association between vaccine status and physical or mental health (S5 Table in S1 File). In sensitivity analyses, T scores were close to the US population norms pre-COVID-19 (S6 Table in S1 File). Groups 1 (Ab+/RNA+) and 3 (Ab-/RNA+) had similar T scores except for physical health, which was worse among people with positive serology (50.47 vs. 51.86; p = 0.002;). There were no significant differences between group 1 (Ab+/RNA+) and true controls (Ab-/RNA- and no prior COVID, 4a. For the subset of participants who had their antibody tests done within 3 months of the RNA test, there was no difference in fair or poor physical or mental health between Group 1 (Ab+/RNA+) and 3 (Ab-/RNA+) groups (S7 Table in S1 File). Adjustment for comorbidity index did not result in substantively different results (S8 Table in S1 File). Among participants from one site in whom we identified a diagnosis of long COVID in the electronic health record, the mean number of symptoms reported 8.07 (SE = 1.07) compared with 4.77 (SE = 0.14; p-value: 0.002) for those without a diagnosis.

**Fig 3:**
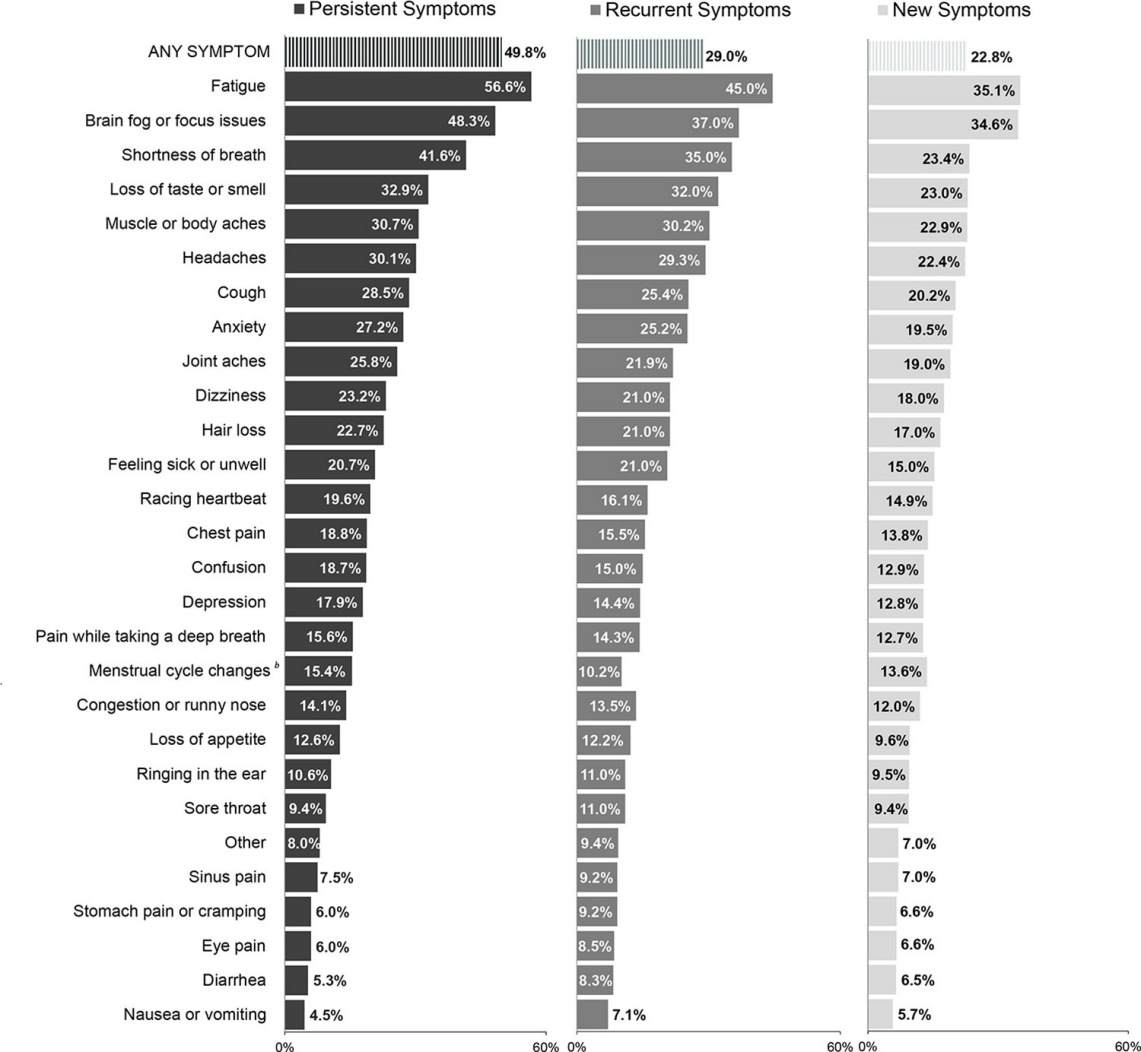
Among participants who reported COVID-19, weighted percentage who reported any persistent, recurrent, or new symptom and percentage of those with specific symptoms, using symptom checklists. ^a^Symptoms not mutually exclusive. ^b^Reported in menstruating women.

Among people who had prior laboratory-confirmed or reported COVID-19, 49.8%, 29.1%, and 22.8% reported any persistent, recurrent, and new symptoms, respectively, using a symptom checklist (Fig 3). Among people who had persistent symptoms consistent with long COVID, fatigue (56.6%), brain fog (48.3%), and dyspnea (41.6%) were the most common. A higher percentage of Group 1 (Ab+/RNA+; 56.9%) reported any persistent symptoms than the other groups (Ab+/RNA- or missing: 47.4%; Ab-RNA+: 54.7%; and Ab-/RNA- and prior COVID: 44.6%) (S9 Table in S1 File); results were similar for new and recurrent symptoms (S10 and S11 Tables in S1 File).

## Discussion

In this multi-site, population-based study, we identified no differences in symptoms and health domains consistent with long COVID based on antibody status among people with laboratory evidence of prior COVID-19. In contrast to prior studies showing an association between antibody response and long COVID [22,47,48], our findings suggest that, among people with prior infection, serologic status using commercially available antibody tests in use at the time this study was completed were not associated with long COVID symptoms, as assessed a mean of 15 months after infection. As a result, in this study, serologic status among people with known infections could not be used to predict symptoms of long COVID.

In contrast to serologic status, RNA positivity was associated with worse physical health, cognitive function, fatigue, palpitations, and headaches affecting work function. These findings were confirmed in sensitivity analyses. Prior studies have also indicated that patients who have had COVID-19 experience problems with cognitive function [9,49,50], fatigue [9,16,20,22,49,51], palpitations [17], and headaches [16,52], supporting the validity of our measures. Our results are consistent with prior estimates that between 30% and 55% of patients report long COVID symptoms [8,21].

For the health domains with general population norms, group scores were unexpectedly close to pre-COVID-19 US population means. These findings may be explained by a substantial symptom burden in the general population. In a study of long COVID using EHR diagnoses, Horberg et al. found a substantial burden of disease in COVID-19 RNA negative controls [20].

This study’s results should be interpreted in the context of its limitations. While this was a population-based sample included English and Spanish speakers from two states, and allowed for electronic and mail responses, we still had lower participation among Spanish-speaking than English-speaking individuals, and our study participants may not be generalizable to the US population. Surveys allowed us to address concerns about the limited capture of long COVID in the EHR, and obtain information about prior COVID-19 infections without testing [53], but we also identified some inconsistencies between self-report and testing results that suggest a subset of patients may not fully understand their laboratory results. We sought to minimize recall bias [53] by using current and recent symptoms for primary analyses, but the length of time since the initial testing may mean some symptoms waned. Prolonged time between RNA testing and serology may have resulted in waning antibody response, and we measured antibody status rather than levels. Antibody responses may vary over time based on viral evolution and improved tests. Some individuals may have had serology testing due to long COVID symptoms or other informative clinical indications. Participants who obtained a positive serology result could have over-reported symptoms relative to people who obtained a negative result. Furthermore, patients were not evaluated over time, did not undergo clinical evaluation, and reported symptoms may not represent long COVID, or could be related to other medical conditions, environmental factors, behaviors, or stressors associated with the COVID-19 pandemic. Most participants completed the survey during the predominance of Omicron. Of the participants who reported having had COVID-19, 13.9% reported having had COVID-19 during the Omicron phase, when home testing was more common. Individuals with long COVID may have been more likely to participate than those without. Despite these limitations, this study occurred during a unique time frame during which serology could be collected prior to exposure to vaccination and our sample was recruited to represent a generalizable sample.

While our study confirmed differences in long COVID based on RNA status, serologic test results were not associated with long COVID among people with laboratory-confirmed COVID-19. Further research is needed to develop other approaches to identify and treat long COVID.

## Supporting information

S1 File COVID long haul supporting information revised. (DOCX)

S2 File STROBE checklist long COVID update 032024 final. (DOCX)

S3 File (DOCX)
