# Supplementary material for: Assessing the association between antibody status and symptoms of long COVID: A multisite study
Source: PLoS One. 2024 Jun 6;19(6):e0304262. doi: 10.1371/journal.pone.0304262 (PMC11156415; doi:10.1371/journal.pone.0304262)
Supplement: S1 File — (DOCX) [file pone.0304262.s001.docx]

**Supporting information to: Assessing the association between antibody status and symptoms of long COVID: a multisite study**

Ingrid A. Binswanger, Darryl Palmer-Toy, Jennifer C. Barrow, Komal J. Narwaney, Katia J. Bruxvoort, Courtney R. Kraus, Jason A. Lyons, Jessica A. Lam, Jason M. Glanz

**S1 Fig. Study design**.
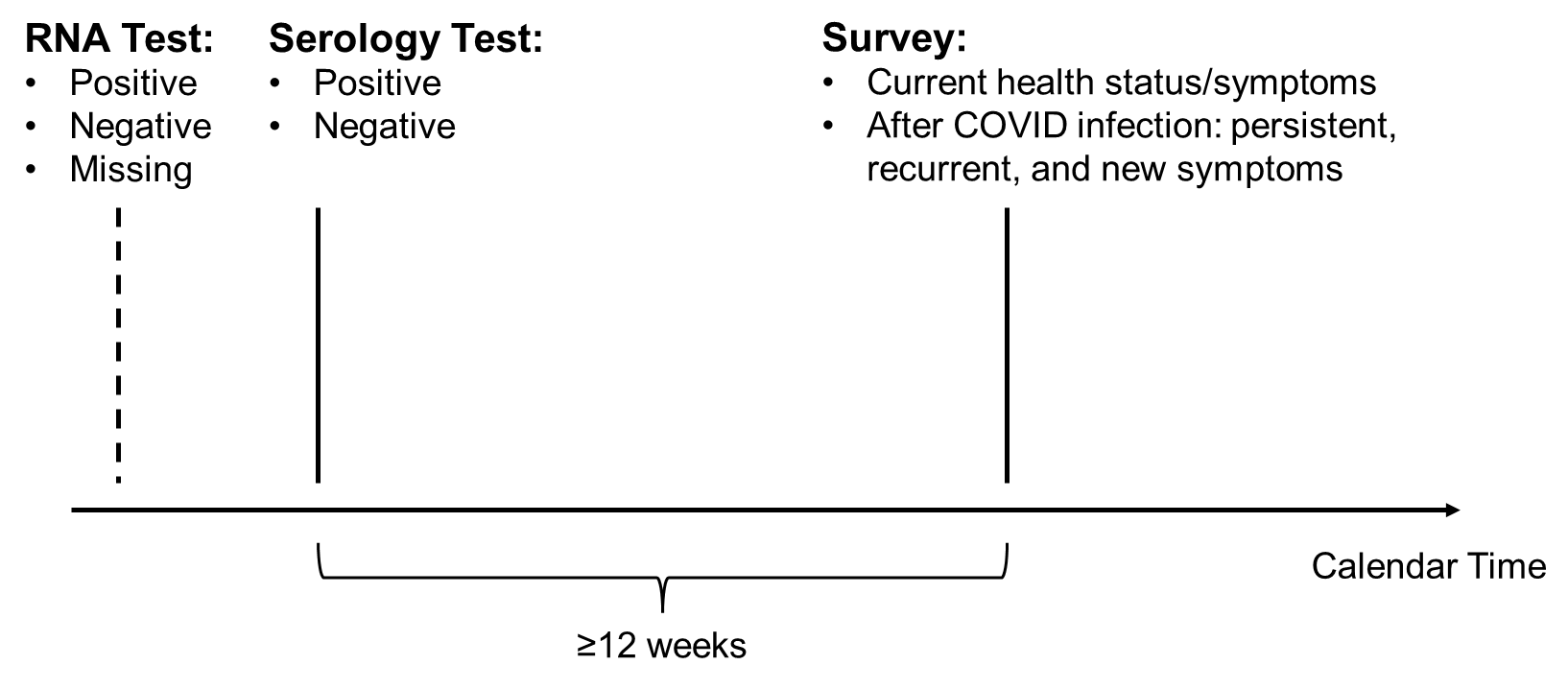


**S1 Table. Survey instrument scales and items and sources used as outcomes in the analysis.^1-7^**

| **Health domain or Symptom** | **Source of scale or item** | **No. of questions in survey** | **Reference periods** |
| --- | --- | --- | --- |
| Symptom checklist | Developed by investigators | 31 | Persistent, Recurrent, New |
| Quality of life | PROMIS Global Health v1.2 – Physical Health | 2 | Current |
|  | PROMIS Global Health v1.2 – Mental Health | 2 | Current |
| Gastrointestinal symptoms | PROMIS Nausea and Vomiting v 1.0 | 4 | 7 days |
|  | PROMIS Diarrhea v 1.0 | 6 | 7 days |
| Anxiety and depression | PROMIS Anxiety v 1.0 | 4 | 7 days |
|  | PROMIS Depression v 1.0 | 4 | 7 days |
| Cognition | PROMIS Cognitive Function_v2.0 | 4 | 7 days |
| Pain | PROMIS Neuropathic Pain Quality v 2.0 | 5 | 7 days |
|  | PROMIS Nociceptive Pain Quality v 2.0 | 5 | 7 days |
|  | PROMIS Pain Interference v 1.1 | 4 | 7 days |
| Dyspnea | PROMIS Dyspnea Characteristics v 1.1 | 1 | 7 days |
| Fatigue | PROMIS Fatigue v 1.0 | 4 | 7 days |
| Sense of smell and taste | University College London Patient Led Research Questionnaire -- Long COVID | 2 | 7 days |
| Palpitations | Health and Quality of Life Outcomes for Arrhythmia | 3 | 7 days |
| Sleep | PROMIS Sleep Disturbance v 1.0 | 4 | 7 days |
| Lightheadedness | Scales for Outcomes in Parkinson’s Disease Autonomic Dysfunction | 3 | 4 weeks |
| Chest pain | Seattle Angina Questionnaire | 1 | 4 weeks |
| Headache interference | ID-Migraine Questionnaire | 1 | 4 weeks |
| Hair loss | Hamilton-Norwood and Ludwig scales | 1 | 4 weeks |
| Menstrual irregularities | Developed by investigators | 1 | 3 months |

Abbreviations: PROMIS=Patient-Reported Outcomes Measurement Information System**S2 Table.** **Differences between study participants and non-participants/excluded participants based on data derived from electronic health records.^a,b^**

|  | **Participants** | **Non-participants** |
| --- | --- | --- |
| **Overall, n (%)** | 3946 (25.5) | 11545 (74.5) |
| **Kaiser Permanente Site, n (%)** |  |  |
| Southern California | 2260 (57.3) | 8763 (75.9) |
| Colorado | 1686 (42.7) | 2782 (24.1) |
| **Survey version, n (%)** |  |  |
| English | 3773 (95.6) | 9931 (86.0) |
| Spanish | 173 (4.4) | 1614 (14.0) |
| **Recruitment group, n (%)** |  |  |
| (1) Ab+ / RNA+ | 1537 (39.0) | 3676 (31.8) |
| (2) Ab+ / RNA- or none | 1338 (33.9) | 4038 (35.0) |
| (3) Ab- / RNA+ | 379 (9.6) | 1297 (11.2) |
| (4) Ab- / RNA- | 692 (17.5) | 2534 (22.0) |
| **Age, mean (SD), years^c^** | 52.1 (15.6) | 48.4 (15.3) |
| **Female, n (%)** | 2695 (68.3) | 6913 (59.9) |
| **Ethnicity, n (%)** |  |  |
| Hispanic | 1120 (28.4) | 5044 (43.7) |
| Non-Hispanic | 2530 (64.1) | 5582 (48.4) |
| Unknown | 296 (7.5) | 919 (8.0) |
| **Race, n (%)** |  |  |
| African American | 149 (3.8) | 579 (5.0) |
| Asian/Pacific Islander | 254 (6.4) | 814 (7.0) |
| Native American | 16 (0.4) | 63 (0.6) |
| White | 2874 (72.8) | 7222 (62.6) |
| Multiple | 79 (2.0) | 256 (2.2) |
| Other race | 77 (2.0) | 129 (1.1) |
| Unknown | 497 (12.6) | 2482 (21.5) |
| **Modified Charlson comorbidity index, mean (SD)** | 0.7 (1.3) | 0.7 (1.4) |

Numbers indicate column percentages.

^a^Assessed using electronic health record data.

^b^p <0.05 for all comparisons except modified Charlson comorbidity index.

^c^Assessed on the first survey invitation date.

**S2 Fig. Timing of self-reported COVID-19 among participants who reported prior COVID-19 at both sites.**


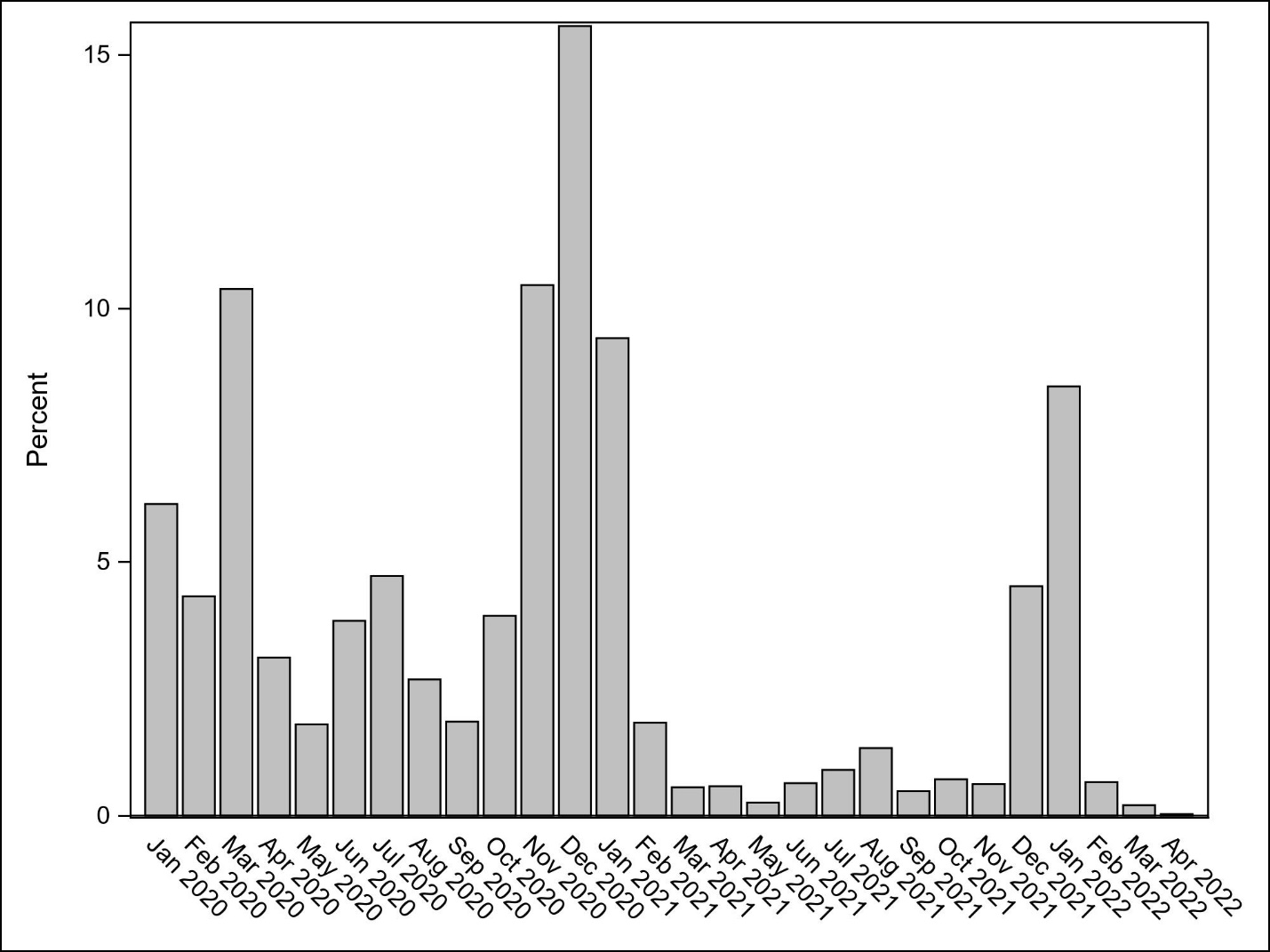


**S3 Table.** **Weighted percentage of people reporting symptoms by group.**

|  | **Adjusted Group Proportion (SE)** | | |
| --- | --- | --- | --- |
| **Domain** | **Ab+/RNA+ (1)** | **Ab+/RNA- or missing RNA (2)** | **Ab-/RNA- reporting prior COVID-19 (4b)** |
| Fair/poor physical health^a, e^ | 18.47 (0.02) | 11.33 (0.01) | 18.35 (0.03) |
| Fair/poor mental health^a^ | 8.79 (0.01) | 7.88 (0.01) | 11.49 (0.02) |
| Cognitive function impairment^a,f^ | 33.05 (0.02) | 30.76 (0.02) | 43.38 (0.03) |
| Fatigue^a,e,f^ | 31.10 (0.02) | 24.90 (0.02) | 38.96 (0.03) |
| Anxiety^a^ | 31.82 (0.02) | 29.31 (0.02) | 33.75 (0.03) |
| Depression^a^ | 21.60 (0.02) | 22.19 (0.02) | 23.13 (0.03) |
| Pain interference^a,e,f^ | 27.00 (0.02) | 20.01 (0.02) | 33.12 (0.03) |
| Nausea Vomiting^b^ | 19.45 (0.02) | 17.21 (0.02) | 24.22 (0.03) |
| Diarrhea^b,e,f^ | 24.24 (0.02) | 18.70 (0.02) | 31.82 (0.03) |
| Sleep disturbance^b,e,f^ | 21.03 (0.02) | 16.66 (0.02) | 26.52 (0.03) |
| Neuropathic pain^b,e^ | 23.03 (0.02) | 18.17 (0.02) | 26.91 (0.03) |
| Nociceptive pain^b,e,f^ | 21.87 (0.02) | 16.55 (0.02) | 30.31 (0.03) |
| Palpitations^c^ | 30.77 (0.02) | 27.61 (0.02) | 35.59 (0.03) |
| Light-headedness^c^ | 38.02 (0.02) | 35.65 (0.02) | 44.04 (0.03) |
| Dyspnea^d,e^ | 37.37 (0.02) | 30.39 (0.02) | 41.13 (0.03) |
| Chest pain^d,e^ | 16.52 (0.02) | 13.29 (0.01) | 17.90 (0.03) |
| Pain^d,e,f^ | 40.72 (0.02) | 34.44 (0.02) | 49.67 (0.03) |
| Loss of smell^d,f^ | 4.59 (0.01) | 6.0 (0.01) | 7.86 (0.02) |
| Loss of taste^d,f^ | 5.38 (0.01) | 6.02 (0.01) | 8.33 (0.02) |
| Hair loss^d^ | 14.54 (0.02) | 14.39 (0.02) | 17.68 (0.02) |
| Headaches limiting work^d,f^ | 21.22 (0.02) | 18.68 (0.02) | 29.01 (0.03) |
| Menstrual irregularities^d, g^ | 42.14 (0.04) | 39.23 (0.04) | 47.15 (0.06) |
| No symptoms^e,f^ | 18.82 (0.02) | 23.35 (0.02) | 12.13 (0.02) |

^a^PROMIS T-score (using PROMIS cut-offs): Physical health, mental health: fair/poor versus good/very good/excellent; cognitive function, fatigue, anxiety, depression, pain interference: mild/moderate/severe versus normal.

^b^PROMIS T-score (using cut-offs from data): Nausea/vomiting, diarrhea, sleep disturbance, neuropathic pain, nociceptive pain: 75^th^ percentile.

^c^More than 1 question asked, analyzed if answered yes to any of the questions in that domain.

^d^Single question asked for the domain and analyzed.

^e^p < 0.05 comparing groups 1 and 2.

^f^p < 0.05 comparing groups 1 and 4b.

^g^Reported in menstruating women.

**S4 Table.** **Weighted percentages for domains comparing those who were hospitalized with COVID-19 versus not among participants who reported COVID-19.**

|  | **Adjusted group percentage (SE)** | |  |
| --- | --- | --- | --- |
| **Domain** | **Hospitalized** | **Not hospitalized** | **p-value** |
| Physical health^a^ | 39.33 (0.04) | 14.05 (0.01) | <.0001 |
| Mental health^a^ | 16.69 (0.03) | 8.62 (0.01) | 0.0006 |
| Cognitive function^a^ | 46.38 (0.04) | 35.19 (0.02) | 0.009 |
| Fatigue^a^ | 46.88 (0.05) | 30.35 (0.02) | 0.0003 |
| Anxiety^a^ | 44.08 (0.05) | 30.78 (0.02) | 0.002 |
| Depression^a^ | 30.53 (0.04) | 21.66 (0.02) | 0.01 |
| Pain interference^a^ | 41.50 (0.04) | 25.37 (0.02) | <.0001 |
| Nausea Vomiting^b^ | 29.53 (0.04) | 19.62 (0.02) | 0.005 |
| Diarrhea^b^ | 30.65 (0.04) | 24.63 (0.02) | 0.10 |
| Sleep disturbance^b^ | 30.87 (0.05) | 20.61 (0.02) | 0.01 |
| Neuropathic pain^b^ | 34.15 (0.05) | 21.87 (0.02) | 0.002 |
| Nociceptive pain^b^ | 37.00 (0.05) | 21.55 (0.02) | 0.0001 |
| Palpitations^c^ | 35.87 (0.04) | 31.53 (0.02) | 0.30 |
| Light-headedness^c^ | 41.90 (0.04) | 39.39 (0.02) | 0.56 |
| Dyspnea^d^ | 56.00 (0.04) | 34.94 (0.02) | <.0001 |
| Chest pain^d^ | 19.73 (0.04) | 15.70 (0.02) | 0.20 |
| Any pain^d^ | 58.78 (0.04) | 40.36 (0.02) | <.0001 |
| Loss of smell^d^ | 2.56 (0.01) | 6.65 (0.01) | 0.002 |
| Loss of taste^d^ | 4.55 (0.01) | 6.85 (0.01) | 0.10 |
| Hair loss^d^ | 20.47 (0.03) | 14.91 (0.02) | 0.07 |
| Headaches limiting ability to work^d^ | 33.45 (0.05) | 22.15 (0.02) | 0.006 |
| Menstrual irregularities^d, g^ | 53.12 (0.14) | 42.91 (0.04) | 0.47 |
| No symptoms | 9.42 (0.02) | 18.16 (0.01) | 0.008 |

Abbreviations: PROMIS=Patient-Reported Outcomes Measurement Information System

^a^PROMIS T-score (using PROMIS cut-offs): physical health, mental health: fair/poor versus good/very good/excellent; cognitive function, fatigue, anxiety, depression, pain interference: mild/moderate/severe versus normal.

^b^PROMIS T-score (using cut-offs from data): Nausea/vomiting, diarrhea, sleep disturbance, neuropathic pain, nociceptive pain: 75^th^ percentile.

^c^More than 1 question asked, analyzed as “yes” if answered yes to any of the questions in that domain.

^d^Single question asked for the domain and analyzed.

^g^Reported in menstruating women.

**S5 Table. Weighted percentage of participants reporting symptoms by survey-reported vaccination status.**

|  | **Adjusted Group Percentage (SE)** | | | **p-value** | |
| --- | --- | --- | --- | --- | --- |
| **Health Domain** | **No vaccine** | **Received at least 1 vaccine** | **Received booster** | **Received at least 1 vaccine vs. no vaccine** | **Received booster vs. no vaccine** |
| Fair/poor physical health^a^ | 18.08 (0.03) | 15.59 (0.02) | 15.04 (0.01) | 0.42 | 0.31 |
| Fair/poor mental health^a^ | 8.65 (0.02) | 10.36 (0.02) | 8.64 (0.01) | 0.50 | 0.99 |

^a^PROMIS T-score (using PROMIS cut-offs): physical health, mental health: fair/poor versus good/very good/excellent.

**S6 Table.** **Adjusted T scores for health domains^a^ by group**.

|  | **T-score (SE)** | | | **p-value** | |
| --- | --- | --- | --- | --- | --- |
| **Health domain** | **(1)**  **Ab+/RNA+** | **(3)**  **Ab-/RNA+** | **(4a)**  **Ab-/RNA-/no prior COVID** | **Group (1) vs. (3)** | **Group (1) vs. (4a)** |
| Physical Health^b^ | 50.47 (0.35) | 51.86 (0.49) | 51.59 (0.59) | 0.002 | 0.06 |
| Mental Health^b^ | 50.48 (0.39) | 50.86 (0.54) | 50.97 (0.63) | 0.45 | 0.44 |
| Cognitive function^b^ | 49.50 (0.46) | 49.58 (0.66) | 51.05 (0.70) | 0.89 | 0.03 |
| Fatigue^c^ | 48.99 (0.51) | 49.18 (0.72) | 47.71 (0.81) | 0.78 | 0.12 |
| Anxiety^c^ | 49.83 (0.44) | 49.20 (0.61) | 51.01 (0.73) | 0.27 | 0.11 |
| Depression^c^ | 47.19 (0.37) | 46.88 (0.54) | 48.16 (0.63) | 0.53 | 0.13 |
| Pain interference^c^ | 48.13 (0.44) | 47.68 (0.60) | 48.05 (0.71) | 0.41 | 0.92 |

^a^Where the centering population is the US general population; measures are from the Patient- Reported Outcomes Measurement Information System; all domains reference the last 7 days except physical health and mental health.

^b^Higher scores, better function.

^c^Higher scores, worse function.

**S7 Table. Weighted percentage of participants reporting symptoms for whom the time between RNA test and antibody test was 3 months or less.**

|  | **Adjusted Group Percentage (SE)** | | **p-value** |
| --- | --- | --- | --- |
| **Health Domain** | **(1) Ab+/RNA+** | **(3) Ab-/RNA+** | **Group (1) vs. (3)** |
| Fair/poor physical health^a^ | 18.14 (0.02) | 12.91 (0.03) | 0.07 |
| Fair/poor mental health^a^ | 9.33 (0.02) | 9.39 (0.03) | 0.98 |

^a^PROMIS T-score (using PROMIS cut-offs): physical health, mental health: fair/poor versus good/very good/excellent.

**S8 Table. Weighted percentage of participants reporting symptoms by group, adjusted for Modified Charlson Comorbidity Index.**

|  | **Adjusted Group Percentage (SE)** | | | **p-value** | |
| --- | --- | --- | --- | --- | --- |
| **Health Domain** | **(1)**  **Ab+/RNA+** | **(3)**  **Ab-/RNA+** | **(4a – true control)**  **Ab-/RNA-/no prior COVID-19** | **Group (1) vs. (3)** | **Group (1) vs. (4a)** |
| Fair/poor physical health^a^ | 17.48 (0.02) | 14.01 (0.02) | 10.48 (0.02) | 0.07 | **0.003** |
| Fair/poor mental health^a^ | 8.70 (0.01) | 8.88 (0.02) | 7.49 (0.02) | 0.90 | 0.54 |
| Cognitive function impairment^a^ | 32.87 (0.02) | 33.31 (0.03) | 24.73 (0.03) | 0.87 | **0.02** |
| Fatigue^a^ | 30.72 (0.02) | 31.41 (0.03) | 23.30 (0.03) | 0.78 | **0.02** |
| Anxiety^a^ | 31.71 (0.02) | 29.45 (0.03) | 35.22 (0.03) | 0.38 | 0.31 |
| Depression^a^ | 21.38 (0.02) | 20.23 (0.02) | 22.58 (0.03) | 0.59 | 0.68 |
| Pain interference^a^ | 26.44 (0.02) | 23.44 (0.03) | 23.31 (0.03) | 0.22 | 0.33 |
| Nausea Vomiting^b^ | 19.24 (0.02) | 20.45 (0.02) | 15.74 (0.03) | 0.56 | 0.20 |
| Diarrhea^b^ | 23.95 (0.02) | 22.72 (0.03) | 20.31 (0.03) | 0.59 | 0.21 |
| Sleep disturbance^b^ | 20.55 (0.02) | 18.00 (0.02) | 15.08 (0.03) | 0.22 | 0.06 |
| Neuropathic pain^b^ | 22.65 (0.02) | 21.54 (0.03) | 18.51 (0.03) | 0.64 | 0.16 |
| Nociceptive pain^b^ | 21.13 (0.02) | 19.03 (0.02) | 23.19 (0.03) | 0.34 | 0.49 |
| Palpitations^c^ | 30.34 (0.02) | 32.08 (0.03) | 21.21 (0.03) | 0.50 | **0.004** |
| Light-headedness^c^ | 37.76 (0.02) | 35.71 (0.03) | 31.54 (0.03) | 0.45 | 0.08 |
| Dyspnea^d^ | 37.02 (0.02) | 35.16 (0.03) | 30.01 (0.03) | 0.50 | 0.05 |
| Chest pain^d^ | 16.33 (0.02) | 15.51 (0.02) | 12.73 (0.02) | 0.67 | 0.15 |
| Any pain^d^ | 40.32 (0.02) | 40.99 (0.03) | 41.36 (0.04) | 0.81 | 0.78 |
| Loss of smell^d^ | 4.61 (0.01) | 5.24 (0.01) | 3.42 (0.01) | 0.54 | 0.42 |
| Loss of taste^d^ | 5.38 (0.01) | 5.20 (0.01) | 3.26 (0.01) | 0.87 | 0.18 |
| Hair loss^d^ | 14.43 (0.01) | 14.12 (0.02) | 11.51 (0.02) | 0.86 | 0.21 |
| Headaches limiting ability to work^d^ | 21.28 (0.02) | 23.34 (0.03) | 14.68 (0.03) | 0.36 | **0.03** |
| Menstrual irregularities^d,e^ | 42.02 (0.04) | 39.13 (0.05) | 45.45 (0.06) | 0.53 | 0.63 |

^a^PROMIS T-score (using PROMIS cut-offs): physical health, mental health: fair/poor versus good/very good/excellent; cognitive function, fatigue, anxiety, depression, pain interference: mild/moderate/severe versus normal.

^b^PROMIS T-score (using cut-offs from data): Nausea/vomiting, diarrhea, sleep disturbance, neuropathic pain, nociceptive pain: 75^th^ percentile.

^c^More than 1 question asked, analyzed as present if participants answered yes to any of the questions in that domain.

^d^Single question asked for the domain and analyzed.

^e^Reported in menstruating women.

**S9 Table.** **Persistent symptoms by group among people who had prior COVID-19.**

|  | **Weighted percentage (SE)** | | | |
| --- | --- | --- | --- | --- |
| **Persistent Symptoms on Symptom Checklist** | **(1)**  **Ab+/RNA+** | **(2) Ab+/RNA-/no RNA** | **(3)**  **Ab-/RNA+** | **(4b)**  **Ab-/RNA- prior COVID^a^** |
| Any symptom | 56.9 (1.5) | 47.4 (1.6) | 54.7 (2.6) | 44.6 (2.9) |
| Fatigue | 54.4 (2) | 46.6 (2.3) | 50.8 (3.6) | 67 (4.2) |
| Brain fog or focus issues | 47.7 (2) | 41.4 (2.3) | 50.5 (3.5) | 53.4 (4.4) |
| Shortness of breath | 42.3 (2) | 34.7 (2.2) | 24.8 (3.1) | 48.2 (4.4) |
| Loss of taste or smell | 33.5 (1.9) | 36 (2.2) | 37.8 (3.5) | 29.2 (4.1) |
| Muscle or body aches | 28.4 (1.8) | 21.3 (1.9) | 21.4 (2.9) | 41.4 (4.4) |
| Headaches | 27.8 (1.8) | 25.1 (2) | 28 (3.2) | 36.6 (4.3) |
| Cough | 25.5 (1.8) | 23.3 (1.9) | 18.3 (2.8) | 36.9 (4.3) |
| Anxiety | 26.5 (1.8) | 22.7 (2) | 22.9 (3) | 31.8 (4.2) |
| Joint aches | 24.9 (1.7) | 18.8 (1.8) | 18.9 (2.8) | 32.9 (4.2) |
| Dizziness | 20.9 (1.6) | 18.5 (1.8) | 17 (2.7) | 30.1 (4.1) |
| Hair loss | 24.7 (1.8) | 21.4 (1.9) | 19.7 (2.8) | 21.9 (3.7) |
| Feeling sick or unwell | 18.8 (1.6) | 14.7 (1.7) | 21.1 (2.9) | 26.8 (3.9) |
| Racing heartbeat | 18.1 (1.6) | 15.7 (1.7) | 14.4 (2.5) | 24.8 (3.9) |
| Chest pain | 15.3 (1.5) | 14 (1.7) | 13.1 (2.4) | 26.9 (4) |
| Confusion | 16.4 (1.5) | 14.8 (1.7) | 13.9 (2.5) | 24.6 (3.9) |
| Depression | 17.9 (1.5) | 16.4 (1.8) | 14.6 (2.6) | 19.5 (3.5) |
| Pain while taking a deep breath | 12.3 (1.3) | 12.1 (1.6) | 8.7 (2.1) | 22.8 (3.8) |
| Menstrual cycle changes^b^ | 18.4 (3.0) | 10.6 (2.5) | 11.1 (3.1) | 17.3 (6.1) |
| Congestion or runny nose | 11.2 (1.3) | 11.9 (1.5) | 6.6 (1.7) | 20 (3.6) |
| Loss of appetite | 11.9 (1.3) | 11.1 (1.5) | 5.5 (1.6) | 15.4 (3.3) |
| Ringing in the ear | 8 (1.1) | 9.1 (1.4) | 8.7 (2.1) | 14.7 (3.2) |
| Sore throat | 7.2 (1.1) | 4.3 (1) | 5 (1.7) | 16 (3.4) |
| Other | 9.7 (1.2) | 8 (1.2) | 15.2 (2.6) | 5.1 (2) |
| Sinus pain | 5.3 (0.9) | 6.3 (1.2) | 4.7 (1.5) | 11 (2.9) |
| Stomach pain or cramping | 3.8 (0.8) | 4.2 (0.9) | 2.4 (1.1) | 10.4 (2.7) |
| Eye pain | 5.3 (0.9) | 4.6 (1.1) | 3.1 (1.2) | 8.4 (2.4) |
| Diarrhea | 5.5 (0.9) | 3.9 (0.8) | 3.7 (1.3) | 6.2 (2.2) |
| Nausea or vomiting | 4 (0.8) | 2.8 (0.8) | 4 (1.4) | 6.2 (2.2) |
| Rash | 2.7 (0.7) | 1.6 (0.5) | 4 (1.4) | 6.2 (2.2) |
| Fever | 2.9 (0.7) | 1.6 (0.6) | 4.3 (1.6) | 5.5 (2) |
| Nosebleed | 2.3 (0.6) | 1.3 (0.6) | 0.7 (0.6) | 2.6 (1.3) |
| Pink eye | 0.9 (0.3) | 1.1 (0.5) | . (.) | 1.8 (1.3) |

^a^Based on the question: “Did you have, or think you had, COVID virus infection?”

^b^Reported in menstruating women.

**S10 Table.** **Recurrent symptoms by group among people who had prior COVID-19.**

|  | **Weighted percentage (SE)** | | | |
| --- | --- | --- | --- | --- |
| **Recurrent symptoms on symptom checklists** | **(1)**  **Ab+/RNA+** | **(2) Ab+/RNA-/no RNA** | **(3)**  **Ab-/RNA+** | **(4b)**  **Ab-/RNA- prior COVID^a^** |
| Any symptom | 32.5 (1.4) | 26.1 (1.4) | 28.1 (2.4) | 28.2 (2.7) |
| Fatigue | 46.6 (2.7) | 35.1 (3) | 39.8 (4.9) | 49.1 (5.6) |
| Brain fog/focus issues | 35.1 (2.6) | 34.8 (3) | 38.8 (4.9) | 39.7 (5.5) |
| Headaches | 31.6 (2.5) | 27.3 (2.8) | 36.7 (4.8) | 43.7 (5.5) |
| Cough | 21.8 (2.3) | 23.4 (2.7) | 21.4 (4) | 49.3 (5.6) |
| Muscle or body aches | 28.5 (2.5) | 24.6 (2.9) | 25.3 (4.3) | 36.2 (5.5) |
| Shortness of breath | 28.3 (2.5) | 24.8 (2.7) | 26.8 (4.4) | 33.4 (5.3) |
| Joint aches | 24.4 (2.4) | 23.2 (2.8) | 20.5 (4.1) | 28.2 (5.1) |
| Congestion or runny nose | 17.5 (2) | 18.2 (2.5) | 20.7 (3.9) | 37.9 (5.4) |
| Feeling sick or unwell | 17.5 (2.1) | 17.8 (2.5) | 26.8 (4.4) | 28.2 (5.1) |
| Sore throat | 13.3 (1.9) | 14.8 (2.3) | 15.9 (3.5) | 33.8 (5.4) |
| Dizziness | 18.2 (2.1) | 20.5 (2.6) | 25 (4.3) | 24.1 (4.7) |
| Anxiety | 19.3 (2.2) | 20.2 (2.7) | 20.5 (4.1) | 22 (4.7) |
| Confusion | 13.2 (1.8) | 16.8 (2.5) | 11.5 (3.3) | 19 (4.4) |
| Loss of taste or smell | 18 (2.1) | 22.6 (2.7) | 6.9 (2.6) | 9.7 (3.3) |
| Racing heartbeat | 18.3 (2.1) | 14 (2.3) | 20.7 (4) | 11.6 (3.6) |
| Hair loss | 16.8 (2.1) | 14.5 (2.3) | 16.1 (3.8) | 11.7 (3.6) |
| Chest pain | 13.5 (1.9) | 11 (2.1) | 15.2 (3.6) | 17.1 (4.2) |
| Depression | 14.3 (1.9) | 11.6 (2.2) | 6.7 (2.7) | 14.7 (4) |
| Fever | 8 (1.5) | 11.2 (2.1) | 9.3 (3) | 17.3 (4.4) |
| Diarrhea | 9.5 (1.6) | 9.3 (1.9) | 11.9 (3.1) | 13.6 (3.8) |
| Sinus pain | 11 (1.7) | 6.9 (1.5) | 6.3 (2.3) | 14.2 (4) |
| Menstrual cycle changes^b^ | 16.8 (4.1) | 8.9 (3.5) | 14.1 (4.7) | 3.2 (2.2) |
| Pleuritic chest pain | 5.9 (1.3) | 10.2 (2.1) | 7.7 (2.5) | 12.8 (3.8) |
| Ringing in the ear | 8.7 (1.5) | 8.6 (1.9) | 10.2 (3.1) | 10.1 (3.3) |
| Stomach pain/cramping | 6.9 (1.4) | 6.8 (1.7) | 9 (2.8) | 13.1 (4) |
| Other | 12.2 (1.8) | 7.3 (1.7) | 12.8 (3.4) | 5.1 (2.3) |
| Eye pain | 7.1 (1.4) | 4.1 (1.4) | 6.1 (2.4) | 12.5 (3.6) |
| Loss of appetite | 10 (1.7) | 11.4 (2.1) | 4.8 (2.1) | 5.1 (2.3) |
| Nausea or vomiting | 5.3 (1.3) | 10.8 (2.1) | 10.1 (3) | 6.2 (2.7) |
| Rash | 6 (1.3) | 3.8 (1.2) | 6.1 (2.4) | 4.4 (2.2) |
| Pink eye | 2.3 (0.8) | 2.5 (1.1) | 0.8 (0.6) | 4.6 (2.5) |
| Nosebleed | 2.8 (0.9) | 1.6 (0.9) | 1.5 (0.9) | 0.5 (0.4) |

^a^Based on the question: “Did you have, or think you had, COVID virus infection?”

^b^Reported in menstruating women.

**S11 Table.** **New symptoms by group among people who had prior COVID-19.**

|  | **Weighted percentage (SE)** | | | |
| --- | --- | --- | --- | --- |
| **New symptoms on symptom checklists** | **(1)**  **Ab+/RNA+** | **(2) Ab+/RNA-/no RNA** | **(3)**  **Ab-/RNA+** | **(4b)**  **Ab-/RNA- prior COVID^a^** |
| Any symptom | 27.1 (1.3) | 20.3 (1.3) | 22.6 (2.1) | 20.9 (2.3) |
| Brain fog/focus issues | 29.3 (2.6) | 27.7 (3.2) | 39 (5.3) | 45.7 (6.2) |
| Fatigue | 32.5 (2.7) | 27.2 (3.2) | 30.1 (5) | 42.3 (6.2) |
| Shortness of breath | 21.7 (2.4) | 21.4 (2.9) | 19 (4.2) | 27.1 (5.5) |
| Anxiety | 18 (2.2) | 20.3 (2.9) | 19.6 (4.3) | 30.7 (5.9) |
| Hair loss | 26.6 (2.6) | 19.5 (2.8) | 17.3 (4.2) | 21.8 (5.3) |
| Joint aches | 25.3 (2.5) | 20.5 (3) | 19.4 (4.4) | 21 (5.2) |
| Muscle or body aches | 22.3 (2.4) | 17.9 (2.8) | 17.9 (4.3) | 19.6 (5) |
| Headaches | 18 (2.2) | 19.6 (2.8) | 17.9 (4.3) | 21.3 (4.9) |
| Racing heartbeat | 18.3 (2.3) | 14.7 (2.6) | 18.1 (4.2) | 22.8 (5.4) |
| Feeling sick or unwell | 13.3 (2) | 13.7 (2.6) | 11.9 (3.6) | 25.3 (5.4) |
| Other | 22.2 (2.4) | 17.4 (2.5) | 22.6 (4.4) | 9.4 (3.6) |
| Depression | 12.3 (1.9) | 14.5 (2.6) | 14.9 (4) | 19.2 (4.8) |
| Dizziness | 12 (1.9) | 14 (2.5) | 11.3 (3.4) | 19.1 (5) |
| Chest pain | 11.9 (1.9) | 7.5 (1.9) | 6.6 (2.9) | 21 (5.2) |
| Menstrual cycle changes^b^ | 19.6 (4.7) | 18.6 (5.0) | 7.5 (4.5) | 7.7 (5.1) |
| Cough | 10.7 (1.8) | 10.9 (2.2) | 7.9 (2.6) | 17.3 (4.6) |
| Confusion | 10.8 (1.8) | 13.3 (2.5) | 11.1 (3.6) | 14.9 (4.5) |
| Ringing in the ear | 10.2 (1.8) | 7.7 (2) | 16.4 (4) | 18.3 (4.8) |
| Loss of taste or smell | 12.4 (1.9) | 14.6 (2.5) | 14.3 (3.9) | 9.7 (3.9) |
| Loss of appetite | 6.5 (1.4) | 11.3 (2.3) | 1.5 (1.3) | 13.1 (4.3) |
| Pleuritic chest pain | 6.1 (1.4) | 7.6 (2) | 3 (1.8) | 15.4 (4.5) |
| Nasal congestion | 7.2 (1.5) | 10.2 (2.2) | 3.8 (2) | 12.2 (4) |
| Eye pain | 6 (1.4) | 7.1 (2) | 0.9 (0.7) | 8.9 (3.6) |
| Sore throat | 5.3 (1.3) | 5.3 (1.6) | 4.7 (2.1) | 10.3 (3.9) |
| Rash | 4.1 (1.1) | 3.6 (1.3) | 8.3 (2.9) | 11.1 (4.2) |
| Sinus pain | 4.6 (1.2) | 3.8 (1.5) | 5.3 (2.3) | 10.7 (3.9) |
| Stomach pain/cramping | 5.7 (1.4) | 5 (1.5) | 5.3 (2.3) | 7.6 (3.2) |
| Diarrhea | 3.5 (1.1) | 5.6 (1.6) | 4.1 (1.8) | 8.5 (3.2) |
| Nausea or vomiting | 3.4 (1.1) | 4.8 (1.5) | 3 (1.8) | 3.9 (2) |
| Nosebleed | 1.7 (0.8) | 3.3 (1.4) | . (.) | 4.2 (2.6) |
| Fever | 2.5 (0.9) | 1.8 (1) | . (.) | 1 (0.7) |
| Pink eye | 1.2 (0.7) | 1.5 (1) | 3 (1.8) | . (.) |

^a^Based on the question: “Did you have, or think you had, COVID virus infection?”

^b^Reported in menstruating women.

**References for supplemental figures and tables**

1. Withers KL, Wood KA, Carolan-Rees G, Patrick H, Lencioni M, Griffith M. Establishing content validity in a novel patient reported outcome measure for cardiac arrhythmia ablation patients. Health Qual Life Outcomes. 2015;13:38.
2. Northwestern University. HealthMeasures: Transforming How Health is Measured. 2023 [cited 2021 Aug 15]. Available from: <https://www.healthmeasures.net/explore-measurement-systems/promis/intro-to-promis/list-of-adult-measures>.
3. Mattos A, Souza JA, Moreira PFF, Jurno ME, Velarde LGC. ID-Migraine questionnaire and accurate diagnosis of migraine. Arq Neuropsiquiatr. 2017;75(7):446-50.
4. Chan PS, Jones PG, Arnold SA, Spertus JA. Development and validation of a short version of the Seattle angina questionnaire. Circ Cardiovasc Qual Outcomes. 2014;7(5):640-7.
5. Visser M, Marinus J, Stiggelbout AM, Van Hilten JJ. Assessment of autonomic dysfunction in Parkinson's disease: the SCOPA-AUT. Mov Disord. 2004;19(11):1306-12.
6. Athena Akrami P. Online Survey on Recovery from COVID-19. 2021 [cited 2021 Aug 15]. Available from: <https://patientresearchcovid19.com/survey2/>.
7. Gupta M, Mysore V. Classifications of Patterned Hair Loss: A Review. J Cutan Aesthet Surg. 2016;9(1):3-12.
